# Does sexual segregation occur during the nonbreeding period? A comparative analysis in spatial and feeding ecology of three *Calonectris* shearwaters

**DOI:** 10.1002/ece3.5501

**Published:** 2019-09-03

**Authors:** Fernanda De Felipe, José M. Reyes‐González, Teresa Militão, Verónica C. Neves, Joël Bried, Daniel Oro, Raül Ramos, Jacob González‐Solís

**Affiliations:** ^1^ Departament de Biologia Evolutiva, Ecologia i Ciències Ambientals Facultat de Biologia Institut de Recerca de la Biodiversitat (IRBio) Universitat de Barcelona Barcelona Spain; ^2^ Centro Okeanos MARE (Marine and Environmental Science Centre) IMAR (Institute of Marine Research) Universidade dos Açores Horta Portugal; ^3^ Institut Mediterrani d'Estudis Avancats, CSIC‐UIB Esporles Spain; ^4^ Centre d'Estudis Avancats de Blanes‐CSIC Blanes Spain

**Keywords:** diet specialization, geolocation, nonbreeding distribution, seabird migration, sexual size dimorphism, stable isotope analyses

## Abstract

Sexual segregation (SS) is widespread among animal taxa, with males and females segregated in distribution, behavior, or feeding ecology but so far, most studies on birds have focused on the breeding period. Outside this period, the relevance of segregation and the potential drivers of its persistence remain elusive, especially in the marine environment, where animals can disperse over vast areas and are not easily observed. We evaluated the degree of SS in spatio‐temporal distribution and phenology, at‐sea behavior, and feeding ecology during the nonbreeding period among three closely related shearwaters: Scopoli's, Cory's, and Cape Verde shearwaters (*Calonectris diomedea, C. borealis*, and *C. edwardsii*, respectively). We tracked 179 birds (92 males and 87 females) from 2008 to 2013 using geolocation‐immersion loggers and collected the 13th secondary remige (molted in winter) for stable isotope analyses as a proxy of trophic level and diet. The global nonbreeding distribution did not differ between sexes for the three species, but one specific nonbreeding area was visited only by males. Cory's shearwater males remained in areas closer to the colony in a larger proportion compared to females and returned earlier to the colony, probably to defend their nests. Males presented a slightly lower nocturnal flying activity and slightly (but consistently) higher isotopic values of δ^13^C and δ^15^N compared to females. These differences suggest subtle sexual differences in diet and a slightly higher trophic level in males, but the extent to which sexual dimorphism in bill size can determine them remains unclear. Our study showed that SS in ecological niche in seabirds can persist year‐round consistently but at a different extent when comparing the breeding and nonbreeding periods. Based on our findings, we propose that SS in these seabird species might have its origin in an ecological specialization derived from the different roles of males and females during reproduction, rather than from social dominance during the nonbreeding period.

## Graphical abstract

In this article, we evaluated the degree of sexual segregation in spatio‐temporal distribution, phenology (through geolocation data), at‐sea behavior (through immersion data), and feeding ecology (through stable isotope analyses) during the nonbreeding period of three closely related shearwaters: the Scopoli's, the Cory's, and the Cape Verde shearwaters (*Calonectris diomedea*, *C. borealis*, and *C. edwardsii*, respectively) for six consecutive years. The global nonbreeding distribution did not differ between males and females of the three species, but the two sexes showed differences in diet. Overall, our study showed that sexual segregation in ecological niche in seabirds can persist year‐round and might have its origin in an ecological specialization derived from different roles of males and females during reproduction rather than from social dominance.

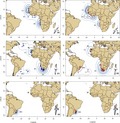

## 1 INTRODUCTION

Sexual segregation (SS) is a widespread behavioral and ecological phenomenon in animal taxa (Rubin & Bleich, 2005). In many terrestrial and aquatic animal species, males and females differ in their spatio‐temporal distribution, at‐sea behavior, and feeding ecology (Catry, Phillips, & Croxall, 2005). SS emerges when males and females make different use of some suitable habitats or food resources, which may ultimately result in intersexual differences in fitness or survival rates, since sexes may be exposed to different conditions or threats (Harrison, Blount, Inger, Norris, & Bearhop, 2011; Marra & Holmes, 2001). Differences in mortality rate among sexes can lead to an imbalance in the sex ratio, with consequences at the population level and broad implications for population dynamics, species conservation, and wildlife management (Durell, Goss‐Custard, & Clarke, 2001; Phillips, Silk, Croxall, Afanasyev, & Bennett, 2005).

Two broad hypotheses have been proposed to explain the general patterns of SS in animals. The social dominance hypothesis suggests that dominant individuals (usually males) tend to exclude subordinates (often females and immatures) from specific areas to access to high‐quality food resources (Gauthreaux, 1978). The ecological specialization hypothesis proposes that habitat segregation arises from sex‐specific preferences, tolerance to ecological factors, or specialization in reproductive roles (Carey, 1996; Ketterson & Nolan, 1983; Morton, 1990; Selander, 1966). Both hypotheses are not mutually exclusive, and their underlying mechanisms can co‐occur and be both cause and consequence (Catry et al., 2005; González‐Solís, Croxall, & Wood, 2000; Shine, 1989).

In birds, sexual differences in migration patterns could be explained by mechanisms related to either of these two general hypotheses, such as competition (related to the social dominance hypothesis) or body size and physiology (both related to the ecological specialization hypothesis; Cristol, Baker, & Carbone, 1999; Gauthreaux, 1982; Ketterson & Nolan, 1983; Myers, 1981). In general, dominant birds tend to remain sedentary and force subordinate individuals to move to areas farther from the breeding grounds to winter (Catry, Dias, Phillips, & Granadeiro, 2013; Gauthreaux, 1982; Pérez, Granadeiro, Dias, Alonso, & Catry, 2013). Furthermore, individuals with a larger body size and better individual physiology (i.e., better thermal tolerance or fasting endurance) would be able to withstand winter in areas closer to the breeding grounds (Ketterson & Nolan, 1976). The tendency of dominant birds to remain resident could also be explained by the arrival time hypothesis, which proposes the earlier arrival of one sex at the end of a migratory journey (related to the ecological specialization hypothesis). According to this hypothesis, the dominant sex tends to be more pressed to arrive earlier at the breeding grounds to gain advantage when competing for better territories or nest sites for breeding (rank advantage hypothesis; Morbey & Ydenberg, 2001) and/or favors more mating opportunities (mate opportunity hypothesis; Morbey & Ydenberg, 2001).

Another indirect mechanism favoring SS is the degree of sexual size dimorphism (SSD) of the species. SSD can contribute to social dominance, as the larger sex is usually the dominant one. Social dominance of one sex can lead to the spatial exclusion of the other at various spatial scales, ranging from subtle differences in microhabitat to disparate geographical distributions (Catry et al., 2005; Staniland, 2006). Nevertheless, SSD can also lead to ecological specialization, due to divergent nutritional and energetic requirements (Main & Coblentz, 1990; Newton, 2008; Ruckstuhl & Neuhaus, 2002), and/or to niche or dietary specialization. The latter occurs when males and females use similar foraging areas but specialize on different prey types due to the morphological differentiation in feeding or locomotion structures (Bearhop et al., 2006; Phillips, McGill, Dawson, & Bearhop, 2011).

Since ecological specialization may arise from differences in the roles of males and females during reproduction, sex‐specific differences in spatio‐temporal distribution and feeding ecology have been widely studied during the breeding period (Elliott, Gaston, & Crump, 2010; Stauss et al., 2012; Thaxter et al., 2009; Weimerskirch et al., 2009). However, studying behavioral and ecological sexual differences out of the breeding period, especially among migratory species, can be challenging due to sampling constraints and limited accessibility to individuals, particularly in the marine environment. As a result, the relevance of SS and the mechanisms of its persistence over the nonbreeding period remain elusive (Alves et al., 2013; Alves et al., 2013; Croxall, Silk, Phillips, Afanasyev, & Briggs, 2005; Müller, Massa, Phillips, & Dell, 2014).

Our capacity to study the spatial and feeding ecology of migratory species during the nonbreeding period has improved considerably in the last decades due to the possibility to combine the deployment of light‐level geolocation devices (geolocators hereafter) and stable isotope analysis (SIA). Geolocators can inform us about the year‐round phenology, movements, distribution, and at‐sea activity patterns (in those cases where loggers are also equipped with an immersion sensor) of a given species. SIA can provide us with information on the feeding and spatial ecology when species feed on isotopically different prey or in areas with distinct isotopic baseline values (Ramos & González‐Solís, 2012). Feathers are metabolically inert after growing and, therefore, their isotopic values reflect the food assimilated by birds during their synthesis (Hobson & Clark, 1992; Ramos & González‐Solís, 2012). Thus, by analyzing feathers molted during the nonbreeding period, we can infer the feeding ecology of birds during such an otherwise inaccessible life stage.

**Figure 1:**
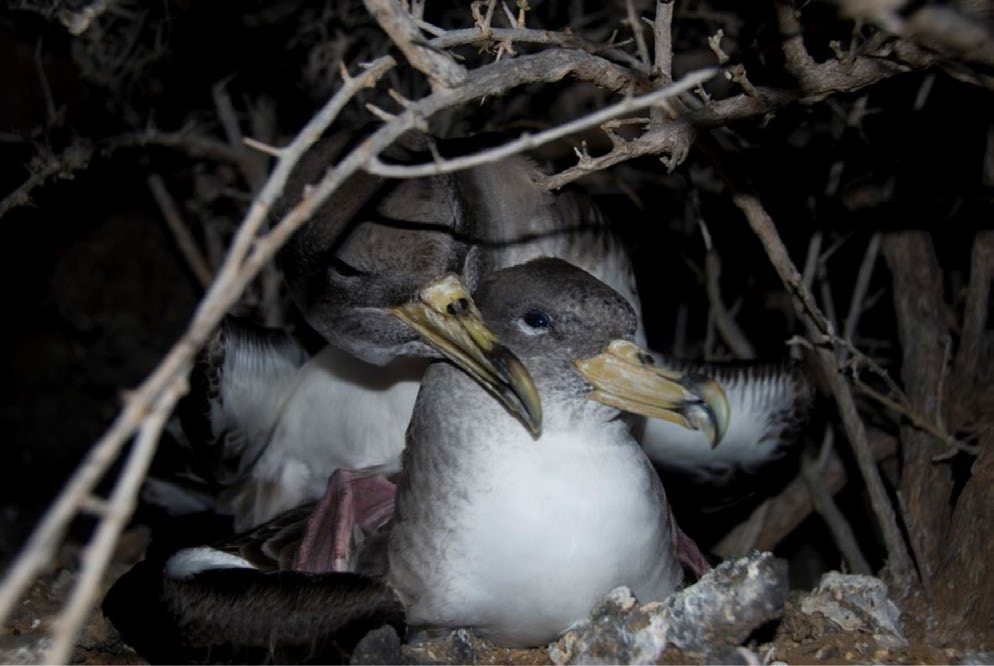
Breeding pair of Cory's shearwaters at Montaña Clara colony, Canary Islands photographed inside their nest in 2008. Photograph by Jacob González‐Solís

*Calonectris* shearwaters are wide‐ranging species, performing long‐distance migrations across ocean basins after the breeding period and spreading over diverse nonbreeding areas (González‐Solís, Croxall, Oro, & Ruiz, 2007; Thibault, Bretagnol, & Rabouam, 1997), thus exposing the individuals to variable environments that can lead to SS in foraging strategies in different ways (Åkesson & Weimerskirch, 2014; Bearhop et al., 2006; Ceia et al., 2012; Phillips, Bearhop, McGill, & Dawson, 2009; Figure 1). These species are relatively well‐studied during the breeding period, and many studies have been done with respect to their SS (Alonso et al., 2014; Werner, Paiva, & Ramos, 2014; Cianchetti‐Benedetti, Catoni, Kato, Massa, & Quillfeldt, 2017; Matsumoto, Yamamoto, Yamamoto, Zavalaga, & Yoda, 2017; Navarro, Kaliontzopoulou, & González‐Solís, 2009; Paiva, Pereira, Ceia, & Ramos, 2017; Ramos, Granadeiro, Phillips, & Catry, 2009a; Ramos, González‐Solís, et al., 2009b). In these species, SS in foraging behavior and feeding ecology may be shaped by annual and seasonal prey availability (Paiva et al., 2017), differences in reproduction duties over the breeding period (Werner et al., 2014; Ramos, González‐Solís, et al., 2009b), and/or could be related to SSD between sexes (Alonso et al., 2014; Cianchetti‐Benedetti et al., 2017). However, while many of these studies did find evidence of sexual differences in foraging and feeding ecology during the breeding period (Alonso et al., 2014; Werner et al., 2014; Cianchetti‐Benedetti et al., 2017; Matsumoto et al., 2017; Paiva et al., 2017; Ramos, González‐Solís, et al., 2009b), many others did not find any clear difference (Navarro, González‐Solís, & Viscor, 2007; Navarro et al., 2009; Ramos, Granadeiro, et al., 2009a). Nonetheless, the degree to which SS in foraging performance continues out of the breeding period is still poorly known in these species (Müller et al., 2014).

In this study, we evaluated the degree of SS in spatial and feeding ecology during the nonbreeding period of three closely related shearwaters: the Scopoli's, Cory's, and Cape Verde shearwaters (*Calonectris diomedea, C. borealis*, and *C. edwardsii*, respectively). In general, we expect that SS in spatial and feeding ecology occurring during the breeding period will not persist during the nonbreeding period, since during this period, seabirds do not have different reproductive roles, are not constrained to return to their nests, and can range for many thousands of kilometers to winter in the most productive areas of the ocean (Bost et al., 2009; Egevang et al., 2010; Shaffer et al., 2006), reducing between‐sex competition and partitioning of food resources (Phillips et al., 2011). Specifically, we aim to test the following three hypotheses: (a) As the larger size of males has been related to a greater involvement in nest defense at the beginning of the breeding period (Werner et al., 2014; Hedd, Montevecchi, Phillips, & Fifield, 2014), we expect males to return to the breeding colonies earlier than females, in accordance with the arrival time hypothesis. (b) Since during the nonbreeding period foraging ranges are not constrained, and shearwaters disperse over wider areas to winter (Shaffer et al., 2006; González‐Solís et al., 2007), we expect that both sexes would share the same nonbreeding areas, and males would not exclude females from areas with high‐quality food resources. (c) Previous studies found differences between sexes in the bill shape and size to be poor predictors of the way males and females (of Cory's shearwaters) exploit the marine environment (Navarro et al., 2009; Ramos, González‐Solís, et al., 2009b). Moreover, between‐sex competition for resources is less intense during the nonbreeding period (González‐Solís et al., 2000). Thus, we expect that males and females would not present differences in their feeding ecology during this period and would feed on similar prey items. Predictions (b) and (c) would refute the social dominance hypothesis for the nonbreeding period, whereas prediction (a) would support the arrival time hypothesis for migratory seabirds. To this end, we evaluated sexual differences during the nonbreeding period of Scopoli's, Cory's, and Cape Verde shearwaters in (a) spatio‐temporal distribution (inferred through geolocation data), (b) at‐sea activity behavior (inferred through immersion data), and (c) feeding ecology (inferred through SIA on one specific feather known to be molted in the winter quarters). Finally, as greater SSD can lead to greater SS (Abouheif & Fairbairn, 1997; Fairbairn, 1997), we also determined the degree of SSD of each species and explored the potential influence of bill size on its feeding ecology.

## 2 MATERIALS AND METHODS

### 2.1 Study species and sampling protocol

Scopoli's shearwater is an endemic breeding species in the Mediterranean Basin, ranging from the Iberian coast to the Adriatic and Aegean Seas (Gómez‐Díaz & González‐Solís, 2007). Cory's shearwaters breed on several islands in the northeast Atlantic Ocean and in a few small colonies in the western Mediterranean Sea (Gómez‐Díaz, González‐Solís, & Peinado, 2009). The Cape Verde shearwater is an endemic breeding species in the Cape Verde Islands (Hazevoet, 1995). Scopoli's and Cory's shearwaters are classified as “Least concern” according to the Red List criteria of the International Union for the Conservation of Nature (IUCN; BirdLife International, 2018), whereas the Cape Verde shearwater is listed as “Near Threatened” due to its restricted breeding distribution (Hazevoet, 2003).

*Calonectris* shearwaters breed mainly on islands and islets, nesting in burrows and crevices. Breeding females lay a single egg per season, and both parents share similar incubation and chick‐rearing duties throughout the breeding season (Granadeiro, Dias, Rebelo, Santos, & Catry, 2006; Thibault et al., 1997). All three species show slight sexual dimorphism in body size, with females being slightly smaller than males in wing length, tarsus length, and bill dimensions and having a less robust shape (Granadeiro, 1993; Massa & Lo Valvo, 1986; Navarro et al., 2009). The breeding phenology of the three species is similar in time: Birds return to the colony from the nonbreeding areas in late February/early March, the laying period begins in the second half of May, and chicks start hatching in mid‐July. Fledglings usually leave the colonies from mid‐October to early November (Granadeiro, 1999; Hazevoet, 1995; Thibault et al., 1997). All three species spend the nonbreeding period in the Atlantic Ocean, mainly in the South Atlantic in areas associated with major upwellings (such as the Benguela and Angola Currents and Brazil Current), and the Canary Current. However, Cory's shearwater can present a broader distribution, with some birds wintering in the North Atlantic and in the southwestern Indian Ocean (González‐Solís et al., 2007; Müller et al., 2014; Petry, Bugoni, & Silva Fonseca, 2000).

In up to five breeding colonies (Table 1), adult birds were captured in their burrows during the breeding period, ringed, and tagged with geolocators. During the subsequent breeding period, we recaptured the birds, retrieved the geolocator, cut the 13th secondary remige (S13 hereafter) for SIA, and we equipped the birds with a new geolocator. During one of the recaptures, we also took a blood sample for molecular sexing and biometric measurements for SSD assessment.

**Table 1:** Summary characteristics of the study colonies and the number of males and females of Scopoli's, Cory's, and Cape Verde shearwaters sampled and tracked in the study period

Species	Breeding colony	Longitude (°)	Latitude (°)	Sampling years	Sample size		Tracks	
					Males	Females	Males	Females
Scopoli's shearwater	Pantaleu islet (Balearic Islands)	2.35	39.57	2009–2013	22	22	35	35
Cory's shearwater	Vila islet (Azores Islands)	−25.17	36.94	2010–2012	12	6	16	9
	Montaña Clara (Canary Islands)	−13.53	29.29	2011–2013	9	11	12	16
	Veneguera (Canary Islands)	−15.78	27.84	2008–2013	44	38	92	76
Cape Verde shearwater	Curral Velho islet (Cape Verde)	−22.78	15.96	2008–2011	5	10	10	14

### 2.2 Molecular sexing

All individuals in the study were molecularly sexed. DNA was extracted from ethanol‐preserved whole blood using a Real Pure genomic DNA extraction kit (Durviz, Spain) following the manufacturer's instructions. Polymerase chain reactions (PCRs) were performed following the method of Fridolfsson and Ellegren (1999), previously used to identify the sex in a large variety of Procellariiform species. Sex determination was based on the detection of a female‐specific locus, CHD1‐W.

### 2.3 Biometric measurements and sexual size dimorphism

We measured five biometric variables on 44, 54, and 16 individuals of Scopoli's, Cory's, and Cape Verde shearwaters, respectively: tarsus length, culmen length, maximum head length (head plus bill length), bill depth at the base, and bill depth at the nostrils. Measurements were taken using digital calipers (±0.01 mm). We assessed the SSD for each biometric measurement and for each study species. SSD index (SSI hereafter) was calculated as:

$$\text{SSI}=\left(\frac{\text{male's average}-\text{female's average}}{(\text{male's average}+\text{female's average})\times 0.5}\right)\times 100.$$

This index is recommended due to its simplicity and because it maintains symmetry around a neutral zero, indicating monomorphy (Storer, 1966). Furthermore, it complies with the convention of positive values in cases where males are the larger sex and negative values in cases where females are the larger ones (Greenwood, 2003). To check the influence of bill SSI on the feeding ecology of the shearwaters, we pooled all individuals measured (*N* = 144) and performed a principal component analysis (PCA) on culmen length, maximum head length, bill depth at the base, and bill depth at the nostrils per each individual. Axis 1 explained a high proportion (92%) of the total variance (Table S1). Therefore, the first principal component scores (scores on axis 1, hereafter referred to as PC1 scores) were used as a proxy of bill size in further statistical analyses (Rising & Somers, 1989).

### 2.4 Geolocation light data

To evaluate whether adult males and females of each species differ spatially in their distribution and/or phenology during the nonbreeding period, we equipped several adult birds of each species with geolocators. The geolocator was attached to a PVC ring with a cable tie, and the ring was put on the leg of the bird. The weight of the geolocators varied from 1.8 g to 4.5 g, depending on the model (models Mk4, Mk9, Mk13, Mk14, Mk18‐H, and Mk19 from the British Antarctic Survey and Mk3005 from Biotrack), corresponding to <1.2% of bird body mass, which is known to have negligible effects on the birds (Carey, 2009; Igual et al., 2005). Overall, we collected information from 70, 221, and 24 geolocators from Scopoli's, Cory's, and Cape Verde shearwaters, respectively, deployed on 182 individuals (Table 1).

Geolocators are devices that record and store ambient light information. The intensity of light is measured every 60 s, and the maximum reading is recorded in 5‐ or 10‐min intervals, depending on the model. Sunset and sunrise times are estimated from thresholds in light curves and are converted into latitudes and longitudes since every location on the planet has a unique combination of time of sunrise and photoperiod in each hemisphere (Hill, 1994), except during the equinoxes. Latitude was derived from day length and longitude from the time of local midday with respect to Greenwich Mean Time. Thus, we assessed two positions of the bird per day with an average accuracy of approximately 186 ± 114 km (Phillips, Silk, Croxall, Afanasyev, & Briggs, 2004). Light data were analyzed visually for every geolocator, using TransEdit and Bird Tracker softwares (British Antarctic Survey, UK), and unrealistic positions were filtered by: (a) removing data during equinoxes due to the inaccuracy of latitude estimation (ca. 20 days before and after the equinoxes); and (b) removing the positions from light curves with obvious interference during the times of sunset or sunrise. We set to 20 the threshold light level considered as the transition between day and night in order to avoid interferences of light during the night and darkness during the day. Before obtaining the trajectory of each bird, sun elevation angles (ranged for −6° to −3°) were calculated based on known positions obtained during a calibration period (approximately 1 week) carried out before the deployments and after recoveries at the breeding colonies. Finally, we smoothed the filtered data twice by interpolating intermediate fixes between successive locations as recommended by Phillips et al. (2004).

To assign each bird (and year) to a single nonbreeding area, we first computed the utilization distribution kernel (KUD hereafter) with previously filtered geolocation data using the function “kernelUD” (R package adehabitat v.1.8.71, Calenge, 2006). We used a bandwidth equivalent to 186 km (~2°, depending on latitude) to account for the average reported error in geolocation (Phillips et al., 2004). Later, we extracted the 50% density contour of the KUD and determined the centroid, using the function “gCentroid” from the R package rgeos (Bivand & Rundel, 2017). We performed a chi‐square test per colony based on the proportion of each sex in each nonbreeding area to determine whether a sexual preference for the use of specific nonbreeding areas existed. In case the 50% density contour of the KUD of a bird was comprised by more than one polygon, the centroid considered for assigning a main nonbreeding area was the one corresponding to the polygon where the bird spent the highest number of days.

In order to determine differences between sexes in the size of the areas used during winter, we first computed KUD using filtered positions for each nonbreeding area and year using a Lambert azimuthal equal‐area projection centered in the centroid of locations to allow area comparability. Next, we calculated the size of the 95% and 50% KUD contours (function “gArea,” package rgeos, Bivand & Rundel, 2017), which were considered to represent the general use and core areas of the wintering distribution, respectively. Lastly, we quantified the amount of overlap between females and males in the general use and core areas of wintering distribution using the “kerneloverlap” function and “HR” method of the adehabitatHR package (Calenge, 2006).

To infer the migratory phenology of our study birds, the filtered positions were inspected visually using Locator software (British Antarctic Survey, UK). Departure dates (from colonies and nonbreeding areas) were defined as the first position outside the cluster of positions of the 10 previous days, when birds shifted behavior and began a rapid directional flight moving away from that cluster. Similarly, arrival dates were defined as the first position of the birds within the cluster of the positions recorded during the days after a rapid directional flight. During the equinoxes, the departure and arrival dates were determined based on the birds' longitude changes (not affected by the equinoxes) as, in most cases, the migratory movement was mainly longitudinal (e.g., Scopoli's shearwater departure from the colony westward toward the Atlantic). In the case of arrival at the breeding colonies occurring during the equinox, we defined the arrival date as the first night the bird spent all night dry (resting at the colony).

### 2.5 At‐sea activity data

The geolocator models used also incorporate a saltwater switch that measures conductivity from immersion in saltwater every 3 s, and combines this information at every 10‐min interval. Given the sampling interval (3 s), the values recorded at the end of each 10‐min period range from 0 (10‐min period in dry mode = no conductivity detected) to 200 (10‐min period in wet mode). These data can be used to infer the behavior of the birds during the nonbreeding season: Complete dryness (0) means that the birds are flying; complete wetness (200) means that the birds are resting (sitting on the sea surface) or diving; and alternate modes between dry and wet (1–199) mean that birds are alternating flying and resting, or could also suggest foraging behavior (Lecomte et al., 2010; Mattern, Masello, Ellenberg, & Quillfeldt, 2015).

To assess whether males and females behave differently at sea during the nonbreeding period, we calculated the night flight index (NFI; Dias, Granadeiro, & Catry, 2012b) of every bird for the period spent in the main nonbreeding area. The ratio of nocturnal/diurnal activity may be associated with prey targeted and thus can provide information about feeding strategies (Dias et al., 2016; Regular, Davoren, Hedd, & Montevecchi, 2010; Spear, Ainley, & Walker, 2007). NFI represents the difference between the proportion of time spent flying during darkness and the proportion of time spent flying during daylight, divided by the highest of these two values, and it varies between −1 (flight activity exclusively during daylight) and 1 (flight activity exclusively during darkness). Moonlight intensity affects activity patterns of shearwaters (Dias, Granadeiro, & Catry, 2012a; Yamamoto et al., 2008). Thus, to control for the influence of moonlight intensity on NFI values, we selected data for an entire lunar cycle (28 days) within the nonbreeding period per individual and year, calculated the NFI for every day of this lunar cycle, and, finally, calculated the mean NFI value per individual and year.

### 2.6 Stable isotope analyses

Stable isotope analyse (SIA) of feathers can be used to study the feeding ecology of seabirds (Hobson, 1999). Feathers become metabolically (and isotopically) inert once fully formed and maintain the isotopic composition of the period and area when they were synthesized, independently of the sampling time (Hobson & Norris, 2008). Knowing the molting patterns of the study species is crucial for SIA, since it allows us to choose which feather to analyze, depending on the period of interest. The molting patterns of Scopoli's and Cory's shearwaters are relatively well known (Alonso, Matias, Granadeiro, & Catry, 2009; Camphuysen & Van Der Meer, 2001; Ramos, Militão, González‐Solís, & Ruiz, 2009c), and they are rather similar between these species. Thus, we assumed it would also be similar for the Cape Verde shearwater. We collected the S13 remige for SIA as this feather is known to be molted at the middle to end of the nonbreeding period in Scopoli's and Cory's shearwaters (since the molt of secondary remiges is asynchronous, and the foci of 12th–16th secondary remiges are the last to be molted; Ramos, Militão, et al., 2009c). In general, δ^15^N increases by 3%–5‰ with each trophic level (DeNiro & Epstein, 1981). δ^13^C also increases with trophic level, although in a smaller proportion (approximately 1‰; Rau, Ainley, Bengtson, Torres, & Hopkins, 1992). The main causes of variations in δ^13^C are differences in photosynthetic biochemistry within and among marine primary producer communities (Farquhar, Ehleringer, & Hubick, 1989; Robinson, 2001). Hence, in marine ecosystems, we can infer the origin of food sources from the δ^13^C gradients that exist between water masses, gradients between inshore/offshore waters, and benthic/pelagic habitats, while δ^15^N values can be used to assess the trophic positions of consumers (Cherel & Hobson, 2007; Newsome, Martinez del Rio, Bearhop, & Phillips, 2007).

Once at the laboratory, feathers were washed in a 0.25 M NaOH solution, thoroughly rinsed twice in distilled water to remove any surface contamination, and dried in an oven at 40°C to constant mass. Afterward, we freeze‐milled all feathers to fine powder in a cryogenic impact grinder (Freeser/mill Spex Certiprep 6750; Spex) operating at liquid nitrogen temperature. We weighed subsamples of 0.30 to 0.32 mg of feather powder and placed them in tin capsules. These samples were oxidized in a Flash EA1112 and TC/EA coupled to a stable isotope mass spectrometer Delta C through a ConFLO III interface (Thermo Finnigan), and, finally, δ^13^C and δ^15^N values were determined. Isotope ratios (*R*) of ^13^C/^12^C and ^15^N/^14^N are expressed conventionally in *δ* units as parts per thousand (‰) according to the following equation:

$$\delta X=\left(\frac{R_{\text{sample}}}{R_{\text{standard}}}-1\right),$$

where *X* (‰) is ^13^C or ^15^N and R are the corresponding ratios ^13^C/^12^C or ^15^N/^14^N related to the standard values. The international standards for SIA are Vienna Pee Dee Belemnite (VPDB) for carbon and atmospheric N_2_ (AIR) for nitrogen. The SIAs were performed at the Serveis Científico‐Tècnics of the University of Barcelona (Spain), where international standards (IAEA CH_7_, IAEA CH_6_, and USGS 40 for C and IAEA N_1_, IAEA N_2_, IAEA NO_3_, and USGS 40 for N) are applied and two standard material samples are inserted every 12 feather samples to calibrate the system and compensate for any drift over time (Böhlke, Mroczkowski, & Coplen, 2003; Böhlke & Coplen, 1995; Coplen et al., 2006; Qi, Coplen, Geilmann, Brand, & Böhlke, 2003; Table S2). The overall measurement error is on average of 0.2‰ for carbon isotopes and 0.3‰ for nitrogen isotopes. All the samples were homogenized by milling them to a fine powder, so we believe that was not necessary to run duplicates. The entire feather analysis methodology was conducted following the “principle of identical treatment” (Bond & Hobson, 2012).

Isotopic data were used to characterize the isotopic niche widths (INW) of each sex through Bayesian statistical ellipses (stable isotope Bayesian ellipses in R—SIBER). We compared INW using a Bayesian estimate of the standard ellipse area (SEAb) to test the probability of a group ellipse of one of the sexes being smaller than the other (Jackson, Inger, Parnell, & Bearhop, 2011). To have a correct estimation of the ellipses, we only considered those nonbreeding areas and years used for a minimum of four birds per sex. Despite the small sample size, it is known that the Bayesian implementation of the ellipse area measurement is less affected by sample size than the convex hull, SEA, and SEAc (Jackson et al., 2011). In addition, using the Bayesian estimation allowed us to provide uncertainty measures (95% credible intervals) around point estimates for the ellipse areas.

### 2.7 Statistical analyses

We performed linear mixed‐effects models (LMMs) to check for sexual differences in the following spatial, phenological, behavioral, and feeding ecological features:

1. Phenological parameters of the migration (represented as day of the year): departure date from the breeding colony (postbreeding migration), days in transit to the nonbreeding areas, total duration of the nonbreeding period, days in the nonbreeding areas, onset of the prebreeding migration, and days in transit returning to the colony and arrival at the breeding colony. These parameters can only be calculated for migratory birds, so we excluded those birds that remain year‐round near the breeding grounds. Nevertheless, we also tested for differences in the date of arrival at the breeding colony between migratory and nonmigratory males of Cory's shearwater and between nonmigratory males and females of Cory's shearwater;
2. Maximum distance traveled from the colony to the centroid of the wintering distribution;
3. Mean size of the core areas of the wintering distribution;
4. NFI values;
5. INW estimated for each nonbreeding area, as indicated by the SEAb values;
6. Values of δ^13^C and δ^15^N assessed on S13 remiges.

Regarding the structure of the models, we always included sex and species as fixed effects. In the models testing for differences in the arrival date at the breeding colony between migratory and nonmigratory Cory's shearwater males, we included migratory behavior (migratory or nonmigratory) and nonbreeding areas as fixed effects. In the models of δ^13^C and δ^15^N, we also included bill size (PC1 scores of bill measurements) as a covariate when testing the effect of the sexual dimorphism on trophic ecology. In the models considering INW, we also included the size of the core area of the wintering distribution within nonbreeding areas as a covariate. Except when modeling INW, bird identity and year were included as random effects to avoid pseudoreplication and nonindependent measurements. When modeling INW, we only included nonbreeding area as a random term, since the INW is calculated by each sex and it is not an individual estimate. When determining the factors affecting migration phenology and the values of δ^13^C and δ^15^N, we also included nonbreeding area as a random term, as well as bird identity and year. Lastly, in the models testing for differences in arrival date at the breeding colony between migratory and nonmigratory males of Cory's shearwater, we included the breeding colony as a random term.

All statistical analyses were performed using R software (version 3.2.5, R Development Core Team, 2010). LMMs were conducted with the function “lmer” (R package lme4, Bates, Mächler, Bolker, & Walker, 2015). To ensure accomplishment of normality and homoscedasticity, we visually inspected Q–Q plots scatter plots of residuals versus fitted values. We created a set of competing models (the first as the full model, including all fixed factors and double interactions) and selected the most parsimonious models, that is, the models that better explain our data using fewer parameters, based on the Akaike's information criterion corrected (AICc) for small sample sizes using the function “dredge” (R package MuMIn, Kamil, 2017). According to the AICc weight (Burnham & Anderson, 2002), we removed nonsignificant terms from our models. When ∆AICc was <2 between our best models, these models explained the data equally well, thus we could not determine which one was the most parsimonious (Burnham & Anderson, 2002). To address this issue, we performed model averaging using the function “model.avg” (R package MuMIn, Kamil, 2017) of those models with ∆AICc < 2 to obtain estimates for our variables. Finally, we performed post hoc comparisons by calculating the differences between the least‐squares means within fixed factors of our best models using the function “difflsmeans” (R package lmerTest, Kuznetsova, Brockhoff, & Christensen, 2015). Whenever multiple comparisons with the same variables were performed, we applied Bonferroni corrections to calculate the correct statistical significance according to the numbers of tests performed.

## 3 RESULTS

### 3.1 Biometric measurements

Sexual size dimorphism index was generally low. Tarsus of Cory's shearwater presented the lowest value (3.4%), whereas bill depth at the nostrils in Scopoli's shearwater showed the highest value (13.4%). Males were, on average, larger than females for the three species, although values of standard deviations overlapped in some extent (i.e., larger females overlapped in size with smaller males). For the three species, differences were more pronounced in bill measurements than in tarsus or maximum head lengths. Scopoli's shearwaters showed the highest SSI among the study species (Tables S3 and S4).

### 3.2 Spatial ecology

#### 3.2.1 Migratory patterns

When testing for sex and species effects on eight migratory parameters, the most parsimonious LMMs always retained species as explanatory factors, and most models also retained sex (Table S5). No sexual differences were found in the maximum distance traveled from the colony to the centroids of the nonbreeding areas or in the number of days in transit to the nonbreeding areas (Tables S5 and S6). For the rest of variables describing migratory patterns, the two best models explained our data equally well (∆AICc < 2) and, thus, we performed model averaging between them. Males left the colonies in autumn 4 days earlier, on average, than females. The total duration of the nonbreeding period (from departure and until the return to the breeding colony), as well as the number of days in the main nonbreeding areas, was greater for males than for females. Males started the prebreeding migration approximately 1 day earlier than females and arrived about 3 days earlier at the breeding grounds, spending fewer days in transit when returning to the colony (Table S5).

Since some Cory's shearwater individuals from Vila and Veneguera did not migrate and remained in areas close to their colonies, we tested whether the return date to the colony differed between nonmigratory and migratory birds. Most parsimonious LMM retained the migratory behavior, but did not retain nonbreeding areas as an explanatory factor. Nonmigratory males arrived about 23 days earlier at the breeding colonies when compared to migratory males. The random effect bird identity explained a higher proportion of the variance not explained by fixed factors than did year or breeding colony factors (Table 2).

**Table 2:** Linear mixed model testing for potential effects of migratory behavior and nonbreeding area on the arrival date at the breeding colony of male Cory's shearwaters. (a) Structure of the candidate models evaluated to explain our data and their associated measures of information (AICc: Akaike's information criterion corrected; ΔAICc: AICc increments of each model in comparison with the best model; AICc_weight_: AICc weights of each model in relation to the set of candidate models). The most parsimonious model is shown in bold. (b) Mean estimates (and 95% confidence intervals in parentheses) of the fixed effects. (c) Variance (±*SD*) and random variance explained (calculated as the percentage of the variance of each random effect divided by the total variance explained by all random effects) by the random effects. All evaluated models included bird identity, year, and breeding colony as random effects

Date of arrival at the breeding colony of migratory and nonmigratory males of Cory's shearwater			
(a) Fixed factors structure	AICc	∆AICc	AICc_weight_
**Migratory**	**949.5**	**0.0**	**0.702**
Nonbreeding area	952.6	3.1	0.149
Migratory + Nonbreeding area	952.6	3.1	0.149
Constant	972.0	22.4	0.000
**(b) Fixed effects**	**Estimates**		
Migratory males	68.3 (57.6, 79.9)		
Nonmigratory males	46.4 (33.1, 59.1)		
**(c) Random Effects**	**Variance ± *SD***		**Random variance explained (%)**
Individual	98.1 ± 9.9		32.7
Year	12.4 ± 3.5		4.1
Colony	46.4 ± 6.8		15.5
Residual	143.1 ± 12.0		47.7

Some male and female Cory's shearwaters from Veneguera did not migrate and remained in the Canary Current, near the colony. We tested whether sex influenced the date of arrival at the breeding colony in these nonmigratory birds. The most parsimonious LMM retained sex, and nonmigratory males returned to the colony about 5 days earlier than nonmigratory females (Table 3).

**Table 3:** Linear mixed model testing for potential effects of sex on the arrival date at the breeding colony of the nonmigratory Cory's shearwaters from Veneguera. (a) Structure of the candidate models evaluated to explain our data and their associated measures of information (AICc: Akaike's information criterion corrected; ΔAICc: AICc increments of each model in comparison with the best model; AICc_weight_: AICc weights of each model in relation to the set of candidate models). The most parsimonious model is shown in bold. (b) Mean estimates (and 95% confidence intervals in parentheses) of the fixed effects. (c) Variance (±*SD*) and random variance explained (calculated as the percentage of the variance of each random effect divided by the total variance explained by all random effects) by the random effects. All evaluated models included bird identity and year as random effects

Date of arrival to the breeding colony of nonmigratory Cory's shearwater			
(a) Fixed factors structure	AICc	∆AICc	AICc_weight_
**Sex**	**183.7**	**0.0**	**0.837**
Constant	187.0	3.3	0.163
**(b) Fixed effects**	**Estimates**		
Males	39.6 (26.5, 52.7)		
Females	44.6 (28.6, 60.5)		
**(c) Random Effects**	**Variance ± *SD***		**Random variance explained (%)**
Individual	18.8 ± 4.3		5.5
Year	52.7 ± 7.3		15.4
Residual	271.6 ± 16.5		79.2

#### 3.2.2 Wintering distribution

**Figure 2:**
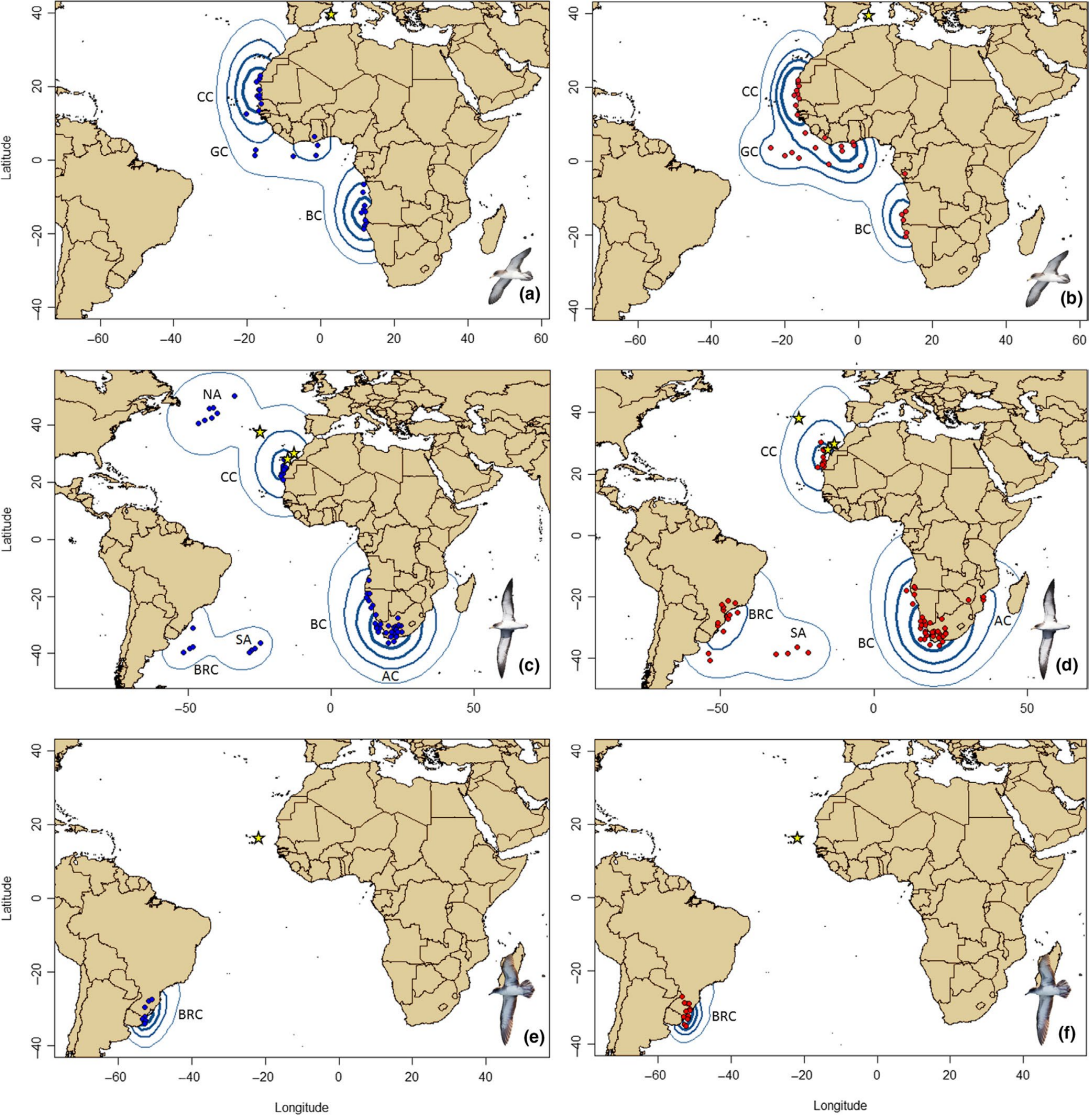
Nonbreeding destinations of males (left, in blue) and females (right, in red) of Scopoli's (a, b), Cory's (c, d), and Cape Verde (e, f) shearwaters: AC = Agulhas Current, BC = Benguela Current, BRC = Brazil Current, CC = Canary Current, GC = Guinea Current, NA = North Atlantic, and SA = South Atlantic. Dots represent the centroid of the nonbreeding position of each individual and year (calculated as averaged coordinates of every 50% UD kernel). UD kernel (25%, 50%, 75%, and 95%, from thicker to lighter blue line contours, respectively) for each sex, and species are also depicted. Yellow stars represent the position of the breeding colonies. Note that, although filters were applied to geolocator data, a percentage of locations occurs on land because of the still relevant influence of the equinoxes. As a result, some individual centroids are on land, although we actually know shearwaters rarely travel inland. Note also that locations over the sea are subject to the same error rate as those on land, although, in this case, it is difficult to recognize

Scopoli's shearwaters wintered in three main areas: the Canary Current (16 males, 12 females), the Guinea and Equatorial Currents (considered as a single area based on their geographical proximity; 7 males, 15 females), and the Angola and Benguela Currents (merged due to geographical proximity and uniformity of stable isotope values of the S13 of the individuals using this area (*t* test: δ^15^N *t*_(16.807)_ = −1.1571 *p* = 0.263; δ^13^C: *t*_(17.270)_ = 1.3909 *p* = 0.182; 12 males, 8 females). No difference was found in the use of the nonbreeding areas by males and females (*χ* ^2^ = 4.3, *df* = 3, *p* = 0.230). For Cory's shearwater, we identified up to six nonbreeding areas: the North Atlantic area (7 males, 0 females), the South Atlantic area (6 males, 4 females), Canary Current (14 males, 10 females), the Angola and Benguela Currents (merged due to geographical proximity and uniformity of stable isotope values of the S13 of the individuals using this area (*t* test: δ^15^N *t*_(3.187)_ = 0.5719 *p* = 0.605; δ^13^C: *t*_(3.048)_ = 0.6692 *p* = 0.551; 78 males, 59 females), the Agulhas Current (9 males, 14 females), and the Brazil Current (2 males, 12 females). We found that male and female Cory's shearwaters did not exploit their nonbreeding areas in a similar manner (*χ* ^2^ = 17.7, *df* = 5, *p* = 0.003). More specifically, no female from Vila islet (Azores) wintered in the Benguela Current or the North Atlantic, while no male from this colony wintered in the Agulhas Current (*χ* ^2^ = 19.2, *df* = 4, *p* < 0.001). Moreover, no female from Montaña Clara (Canary Is.) wintered in the Canary Current or the South Atlantic, while no males wintered in the Agulhas and Brazil Currents (Table S7; *χ* ^2^ = 9.7, *df* = 4, *p* = 0.052). All Cape Verde shearwater individuals wintered in the Brazil Current throughout the study period (10 males, 14 females; Figure 2).

Regarding the size of the core areas of the wintering distribution, most parsimonious LMM retained sex as explanatory factor (Table S9). In general, females used a greater core area than males (mean_males_ = 618,646 km^2^ [365,730−871,560], mean_females_ = 806,545 km^2^ [553,630−1,059,460]). For most years and nonbreeding areas, females of Scopoli's and Cory's shearwaters globally used a greater core area than males within each nonbreeding area. In contrast, males of Cape Verde shearwater showed larger core areas in the Brazil Current during the two years studied (Table S8). Finally, both sexes showed a high degree of overlap in their general use areas (95% Kernel density contours) and in the core areas (50% Kernel density contours) for most nonbreeding areas and years (Table S10).

#### 3.2.3 At‐sea behavior

Night flight index revealed differences among sexes and species. Our model suggested that the females of Scopoli's and Cape Verde shearwaters tended to be more active during the night than males. Cory's shearwaters presented the opposite pattern, and, overall, this species was more active at night than the other two (Table S11).

### 3.3 Trophic ecology

#### 3.3.1 Stable isotope analysis

**Figure 3:**
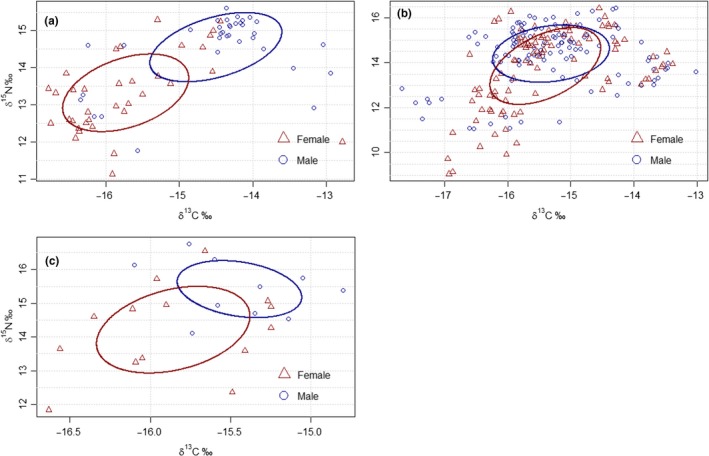
Stable isotope values of δ^13^C and δ^15^N of the 13th secondary remiges (S13) of Scopoli's (a), Cory's (b), and Cape Verde (c) shearwaters for all the study years (2008–2013). The area of the standard ellipses (SEAc) used to compare isotopic niches are represented by solid lines (ellipses; see Jackson et al., 2011). Males are denoted in blue and females in red

Overall, the S13 of the males of the three species showed slightly higher values of δ^13^C (mean estimates_males_ = −15.6 [−16.2, −15.1]) and δ^15^N (mean estimates_males_ = 14.0 [12.8, 15.3]) than in females (mean estimates_females_ = −15.8 [−16.3, −15.3]) and 13.5 [12.3, 14.7] for δ^13^C and δ^15^N values, respectively; Figure 3 and Table 4).

**Table 4:** Linear mixed model testing for potential effects of bill size (residuals of the linear regression of PC1 scores as function of sex) and species in the stable isotope values of δ^13^C (A) and δ^15^N (B). (a) Structure of the candidate models evaluated to explain our data and their associated measures of information (AICc: Akaike's information criterion corrected; ΔAICc: AICc increments of each model in comparison with the best model; AICc_weight_: AICc weights of each model in relation to the set of candidate models). The most parsimonious models and those models with ∆AICc < 2 are shown in bold. (b) Results of the mean estimates (and 95% confidence intervals in parentheses) with adjusted SE obtained after performing model averaging between the best‐supported models with ∆AICc < 2. (c) Relative variance importance of the fixed effects obtained from model averaging. All the performed models included bird identity, year, and nonbreeding area as random effects

δ^13^C				δ^15^N			
(a) Fixed factors structure	AICc	∆AICc	AICc_weight_	(a) Fixed factors structure	AICc	∆AICc	AICc_weight_
**Sex + Species +Sex:Species**	**330.1**	**0.0**	**0.647**	**Sex + Species +Sex:Species**	**462.2**	**0.0**	**0.283**
**Sex + Species + Bill size + Sex:Species**	**331.9**	**1.8**	**0.260**	**Sex + Species +Bill size + Sex:Bill size**	**462.6**	**0.4**	**0.228**
Sex + Species +Bill size + Sex:Species + Sex:Bill size	334.0	3.9	0.091	**Sex + Species**	**463.0**	**0.8**	**0.191**
Sex + Species	343.2	13.1	0.001	**Sex + Species +Bill size + Sex:Species**	**463.4**	**1.3**	**0.150**
Sex + Bill size	344.1	14.0	0.001	Sex + Species +Bill size	464.6	2.4	0.086
Sex + Species +Bill size	345.4	15.3	0.000	Sex + Species +Bill size + Sex:Species + Sex:Bill size	465.3	3.1	0.059
Sex + Bill size + Sex:Bill size	345.5	15.4	0.000	Species + Bill size	473.0	10.8	0.001
Sex	345.9	15.8	0.000	Species	479.3	17.2	0.000
Sex + Species +Bill size + Sex:Bill size	346.5	16.4	0.000	Sex + Bill size	506.6	44.5	0.000
Species + Bill size	351.8	21.7	0.000	Sex + Bill size + Sex:Bill size	508.5	46.4	0.000
Species	365.1	35.0	0.000	Sex	538.4	76.2	0.000
Constant	368.4	38.3	0.000	Constant	554.7	92.5	0.000
Bill size	425.2	95.1	0.000	Bill size	596.3	134.1	0.000
**(b) Fixed effects**	**Estimates**			**(b) Fixed effects**	**Estimates**		
Males	−15.6 (−16.2, −15.1)			Males	14.0 (12.8, 15.3)		
Females	−15.8 (−16.3, −15.3)			Females	13.5 (12.3, 14.7)		
Scopoli's shearwater	0.7 (0.3, 1.2)			Scopoli's shearwater	−0.2 (−1.0, 0.5)		
Cape Verde shearwater	0.8 (−0.1, 1.6)			Cape Verde shearwater	4.9 (3.5, 6.2)		
Females:Scopoli's shearwater	−0.9 (−1.3, −0.5)			Females:Scopoli's shearwater	−0.6 (−1.2, 0.0)		
Females:Cape Verde shearwater	−0.2 (−0.8, 0.3)			Females:Cape Verde shearwater	−0.8 (−1.6, 0.1)		
Bill size	0.1 (−0.1, 0.2)			Bill size	0.1 (−0.1, 0.4)		
				Females:Bill size	−0.2 (−0.3, 0.0)		
**c) Relative variance importance (%)**				**c) Relative variance importance (%)**			
Sex	1.0			Sex	1.0		
Species	1.0			Species	1.0		
Sex:Species	1.0			Sex:Species	0.5		
Bill size	0.3			Bill size	0.4		
				Sex:Bill size	0.3		

The best models for explaining differences in δ^13^C and δ^15^N values included sex, species, their interaction, and bill size. Although all variables were retained in the best models (with exception of the interaction between sex and bill size), the relative importance and significance of sex (1.0) and species (1.0) were higher when comparing with bill size (0.3 and 0.4 for δ^13^C and δ^15^N values, respectively; Table 4). Thus, we performed separate pairwise comparisons, and despite the lack of statistical significance for all the three species, isotopic values were, in general, slightly higher in males (mean estimates = 0.6 [0.3, 0.9], *p* < 0.001 and mean estimates = 0.9 [0.5, 1.4], *p* < 0.001 for δ^13^C and δ^15^N values, respectively). The mean values of δ^13^C (mean estimates = 1.1 [0.7, 1.5], *p* < 0.001) and δ^15^N (mean estimates = 1.1 [0.5, 1.7], *p* < 0.001) were significantly lower in females for Scopoli's shearwater. Isotopic values of Cory's shearwater were similar between sexes (mean estimates = 0.2 [−0.2, 0.6], *p* = 0.272 and mean estimates = 0.4 [−0.1, 1.0], *p* = 0.106 for δ^13^C and δ^15^N values, respectively), and in Cape Verde shearwater, isotopic values were significantly lower in females, only when considering δ^15^N (mean estimates = 0.4 [−0.1, 0.9], *p* = 0.112 and mean estimates = 1.2 [0.4, 1.9], *p* = 0.002 for δ^13^C and δ^15^N values, respectively). Mean δ^15^N values for Cape Verde shearwaters were significantly higher than for the other species (mean estimates = 4.9 [3.5, 6.2], *p* < 0.001; Table S12).

The null model and the one including species best explained the Bayesian estimate of the standard ellipse area (SEAb) values. After performing model averaging, values of the SEAb differed among species—with Cory's shearwater presenting higher values, followed by Scopoli's shearwater—but not between sexes or among the size of core areas of the wintering distribution, with Scopoli's shearwaters showing the broadest isotope niches [mean estimates = 1.5 (0.9, 2.0)] (Tables S13 and S14).

## 4 DISCUSSION

By combining geolocation data and isotopic values of feathers collected over 6 years, we evaluated the SS in spatio‐temporal distribution, at‐sea behavior, and feeding ecology in three closely related seabird species during their nonbreeding period. Migratory males of the three species arrived earlier than females at their breeding grounds, although differences were more subtle than expected (3 days earlier on average). Nonmigratory Cory's shearwater males remained in areas close to the colony in a larger proportion than females and arrived at the breeding colonies earlier than migratory males and both nonmigratory and migratory females, as was found in Catry et al. (2013). Such differences in migratory behavior can be explained by differential roles in reproduction according to the arrival time hypothesis, where the earlier arrival of males confers an advantage in mate acquisition and territory defense (Catry et al., 2013; Hedd et al., 2014; Kokko, Gunnarsson, Morrell, & Gill, 2006). Overall, males and females of the three *Calonectris* species did not differ in their spatial distribution and shared their main nonbreeding areas, except for a specific wintering area of Cory's shearwater located NW of the Azores archipelago, apparently only used by males. Furthermore, males and females did not differ in their spatial distribution when sharing a given nonbreeding area (i.e., at medium geographical scale), which would exclude hypotheses related to social dominance occurring during this period. In all three species, males generally showed greater values of δ^13^C and δ^15^N compared to females, although such differences were not always statistically significant. Given that the distribution within each nonbreeding area did not differ between sexes, this result cannot arise from geographic differences in baseline isotopic levels, but suggests a subtle SS in trophic level and diet. However, we cannot be conclusive in this regard.

### 4.1 Spatio‐temporal segregation between males and females

We found some sexual differences in the timing of migratory movements in the three species and in the use of nonbreeding areas in Cory's shearwater. Only Cory's shearwater males from Vila, and a larger proportion of males than females from Veneguera, remained in areas close to their respective colonies year‐round. Furthermore, males of the three species departed earlier than females from their breeding colonies in autumn at the onset of the postbreeding migration. In most cases, males also arrived earlier than females at the breeding colony, although this difference might vary depending on species, nonbreeding area, and year.

The intersexual differences we found in the nonbreeding distribution of Cory's shearwaters are similar to those previously found for the same species in the Selvagens Islands (Pérez et al., 2013). The social dominance hypothesis could explain these results, with individuals of the larger sex staying closer to the breeding grounds and forcing subordinates to migrate further away. However, in the Veneguera colony (Canary Islands), for which we had a larger sample size, some females also did not migrate and wintered in the Canary Current, near the breeding colony. Furthermore, all areas, except the area NW of the Azores, were shared by males and females and we found no segregation between sexes in the spatial distribution within each nonbreeding area for any of the species we considered. Similarly, Pérez et al. (2013) found no association between body size and the decision to migrate or remain resident in Cory's shearwater. Body size can be ruled out when explaining sexual differences in migration patterns, and our results, therefore, do not support the social dominance hypothesis for explaining the sexual differences observed in the use of the nonbreeding areas.

The arrival time hypothesis could explain both the greater tendency of Cory's shearwater males to remain resident and the slight, but consistent, phenological differences between sexes in the three species we studied. The early arrival of one sex at breeding grounds could be essential to ensure mating opportunities and the acquisition of suitable territories for breeding (Hedd et al., 2014; Ketterson & Nolan, 1983). The earlier arrival of males occurs in many migratory bird species, while the opposite has been observed in only a few sex‐role‐reversed bird species (Kokko et al., 2006; Reynolds, Colwell, & Cooke, 1986). In our study, sexual differences among migratory birds were more subtle than expected, since we found that migratory males arrived at breeding colonies only about 3 days earlier than migratory females on average. However, nonmigratory males arrived approximately 23 days earlier than migratory males, and about 5 days earlier than nonmigratory females, at their respective breeding colonies. Hence, despite the slight difference in the arrival dates among migratory birds, the pattern of males arriving at breeding colonies earlier than females has been consistent, being even more pronounced when males decide to remain resident. We suggest that the differences between migratory males and females are not so pronounced since the birds share the same nonbreeding areas to winter, and the latitudes of nonbreeding areas elected were related to the date of return to the breeding grounds. As previously observed in another study, the farther the shearwaters traveled from the colony, the later they returned to breed in the subsequent breeding period (Müller, Massa, Phillips, & Dell'Omo, 2015). In other studies, females wintered further south/north than males and returned approximately 5–10 days later to breeding colonies (Catry et al., 2005; Müller et al., 2014; Phillips et al., 2005). Furthermore, the earlier departure of males from colonies for the postbreeding migration can be facultative since shearwaters are not territorial at sea and there may be no advantage in arriving at the nonbreeding areas earlier than potential competitors (Kokko, 1999). However, our results are consistent with those of Müller et al. (2015), who have suggested that Scopoli's shearwater males leave the breeding areas earlier than females so they can arrive earlier in the subsequent reproductive season, in a kind of “domino effect” (Briedis et al., 2019). According to the “domino effect,” the timing of one phase of the annual cycle may affect the timing of the subsequent phase (Briedis et al., 2019; Gow et al., 2019), in this case, between postbreeding migration and arrival at the breeding colony for the subsequent reproductive season. Furthermore, although both sexes contribute equally to incubation and chick rearing (Hamer, Schreiber, & Burger, 2002), males tend to spend more time and energy defending the nests at the beginning of the breeding period, which could also explain their earlier arrival (Werner et al., 2014; Hedd et al., 2014). Hence, sex differences in migration distance and timing may be better explained by the different roles in reproduction between males and females (Catry et al., 2005).

### 4.2 Sexual differences in at‐sea behavior and feeding ecology

Our results concerning at‐sea behavior and feeding ecology could be considered consistent with the ecological specialization hypothesis. In the three species, δ^13^C and δ^15^N values of the S13 remige (molted during the nonbreeding season) were slightly higher in males than in females. In seabirds, sexual differences in isotope ratios are often documented during different stages of the breeding period, but do not necessarily remain consistent year‐round (Phillips, Lewis, González‐Solís, & Daunt, 2017; Phillips et al., 2011). The slight differences between sexes in δ^13^C and δ^15^N values found in our study suggest a small dietary segregation between sexes of the three species during the nonbreeding period. These variations may occur due to differences in the metabolic rates between males and females (González‐Solís et al., 2000). However, the extent to which metabolic rates affect species with slight SSD, such as *Calonectris* shearwaters, is poorly known. We also recognize that other factors not considered in this study, such as age, may influence metabolic rates (Alonso et al., 2012). Furthermore, sexual differences in δ^13^C and δ^15^N values could reflect different S13 remige molting strategies among males and females, which occurs during the nonbreeding period (Ramos, Militão, et al., 2009c). Nevertheless, no differences were found in the onset of the molt of the primary remiges of Cory's shearwater males and females during the late chick‐rearing period (Alonso et al., 2009), and further investigation into sexual differences in molting schedules is required. Hence, we argue that at the end of the breeding period, when rearing duties are more relaxed and shearwaters can disperse over wider areas (Shaffer et al., 2006; González‐Solís et al.,^2007^), foraging niches may better reflect intrinsic, sex‐specific feeding preferences that may persist throughout the entire nonbreeding period (Clay et al., 2016).

Differences in carbon isotope ratios are frequently used to determine differences between terrestrial versus marine ecosystems, inshore versus offshore, and pelagic versus benthic food webs (Quillfeldt, McGill, & Furness, 2005). We did not detect a clear spatial segregation between males and females within each nonbreeding area; however, females make use of a greater core area (50% KUD) than males, which may suggest that males forage more efficiently than females (Weimerskirch, Cherel, Cuénot‐Chaillet, & Ridoux, 1997), as females need to forage in a larger area than males to ensure their requirements. Furthermore, higher δ^13^C values in males, particularly in Scopoli's shearwaters, may suggest that males feed more heavily on the benthic prey (with higher δ^13^C values) available at the surface layer in more central areas of the upwelling systems, whereas females feed in more peripheral areas, probably taking advantage of lesser quality food resources, with lower δ^13^C values. The diet of *Calonectris* shearwaters during the nonbreeding period is almost unknown (Barrett et al., 2007; Petry, Krüger, da Silva Fonseca, Brummelhaus, & da Cruz Piuco, 2009). In general, these shearwaters are shallow divers and tend to feed on surface prey during daylight (Cianchetti‐Benedetti et al., 2017; Dias et al., 2012b; Grémillet et al., 2014; McNeil, Drapeau, & Pierotti, 1993), although both Scopoli's and Cory's shearwaters may also forage at night (Dias et al., 2012b; Rubolini, Maggini, Ambrosini, & Imperio, 2015). When in productive waters of nonbreeding areas, birds may make use of the sit‐and‐wait foraging strategy, and food availability may be improved by the activities of subsurface predators and fisheries (Péron et al., 2010; Phillips et al., 2017). The more intense activity at night among female *Calonectris* shearwaters may suggest that they take greater advantage of the diel vertical migration of some mesopelagic fish, crustaceans, and squids (lower trophic level prey characterized by lower δ^15^N values) than males do (Hays, 2003; Spear et al., 2007). In addition, sexual differences in the at‐sea activity patterns and in isotopic values may also result from males exploiting more fishery discards than females (Hobson, Piatt, & Pitocchelli, 1994; Ramos, González‐Solís, et al., 2009b), which are often dominated by inshore benthonic species with higher δ^13^C and δ^15^N values (Bugoni, Griffiths, & Furness, 2011; Hobson et al., 1994). The interactions of Scopoli's shearwaters with longline fisheries increase when the density of the fleet of operating trawlers is lower (and consequently, less discards are available) in the western Mediterranean (Soriano‐Redondo et al., 2016), confirming that fisheries modify the natural way in which seabirds look for resources. Furthermore, the bycatch of Scopoli's shearwaters by longline fisheries in this area is male‐biased, especially during the prelaying period (Cortés, García‐Barcelona, & González‐Solís, 2018).

In sexually dimorphic species, we might expect sexual differences in diet to be the result of different body sizes and, in particular, different sizes of feeding structures, such as the bill in birds (Amadon, 1959; Selander, 1966). Males are larger than females in the three shearwater species considered in this study, particularly with respect to bill size. We found a slight effect of bill measurements on the isotopic differences between sexes, which may suggest that at least some males are capable of feeding on larger prey at higher trophic levels (i.e., with higher δ^15^N values; Cherel & Hobson, 2005). Previous studies conducted on Cory's shearwaters concluded that SSD in bill and wing dimensions was poor predictors of the way males and females exploit the marine environment (Navarro et al., 2009; Ramos, Granadeiro, et al., 2009a). Thus, the role of sexual selection in sexual differentiation in bill size in *Calonectris* shearwaters remains unclear, and results suggest the need to investigate the effect of individual body and bill size once controlled for sex.

## 5 CONCLUSIONS

In summary, Cory's shearwater males preferred to remain closer to the breeding grounds during the nonbreeding period compared to females. Sex‐related differences in several parameters of the migration phenology were also found, with males leaving and arriving earlier than females at the breeding grounds. This could be attributed to differential reproductive roles, in particular to the greater involvement of males in nest defense, rather than to male social dominance. This was supported by the apparent absence of spatial segregation between males and females within all main nonbreeding areas, though this finding should be viewed with some caution due to the lack of fine‐scale spatial resolution of the geolocators. Nevertheless, we observed some differentiation between sexes in nocturnal flight behavior, with males displaying more diurnal flying activity than females in general. This finding was supported by isotopic values, which could reflect differences in feeding preferences and diet composition. However, trophic segregation was not fully supported by the SSD in bill size. Overall, our study showed that SS in ecological niche in seabirds persists year‐round consistently but at a different extent. Based on our findings, and the fact that most of the studies conducted during the breeding period have reported sexual differences in the stable isotope values, we hypothesized that males and females might have evolved in exploiting different ecological niches as a result of an ecological specialization derived from differential reproductive roles (rather than from social dominance), which may persist throughout the annual cycle.

## CONFLICT OF INTEREST

F. De Felipe was supported by a PhD grant from the Coordenação de Aperfeiçoamento de Pessoal de Nível Superior (CAPES—Brazilian government agency; Bex Process 1307/13‐4), J.M. Reyes‐González by a PhD grant from the Spanish government FPU program (AP2009‐2163), T. Militão by a PhD grant (SFRH/BD/47467/2008) from Fundação para a Ciência e Tecnologia (FCT, Portugal), J. Bried and V.C. Neves were supported by FCT (grants SFRH/BPD/36425/2007 and SFRH/BPD/88914/2012) and the MoniAves programme (programme launched by the Regional Environment Directorate from the Azores and coordinated by R.S. Santos), D. Oro was supported by Ministerio de Ciencia, Innovación y Universidades from the Spanish Government (CGL2017‐85210‐P), R. Ramos was supported by Beatriu de Pinós (2010‐BP_A‐00173) and Juan de la Cierva (JCI‐2009‐05426) grants.

## AUTHOR CONTRIBUTIONS

FDF performed the molecular sexing, analyzed geolocator, and derived data, performed the statistical analyses, and drafted the manuscript; JMR‐G conducted fieldwork, analyzed geolocator data, wrote the R code for spatial analyses, and reviewed the draft versions of the manuscript; TM conducted fieldwork, analyzed geolocator data, carried out stable isotope analyses, and reviewed the manuscript; JB, VCN, and DO conducted fieldwork and reviewed the manuscript; RR conducted fieldwork, analyzed geolocator data, provided advice in ecological issues, and reviewed the process of drafting the manuscript; and JG‐S conceived the study, devised structure of the manuscript and data analysis pipeline, got funding, supervised the work, and reviewed the manuscript.

## Data Availability

Tracking data are available at the BirdLife International Seabird Tracking Database (http://www.seabirdtracking.org) under the Dataset IDs: 974, 975, 976, 977, and 983. The data that support the findings of this study are openly available in the University of Barcelona (UB) Digital Repository (http://hdl.handle.net/2445/136898).
