# Supplementary material for: Does sexual segregation occur during the nonbreeding period? A comparative analysis in spatial and feeding ecology of three Calonectris shearwaters
Source: Ecol Evol. 2019 Sep 3;9(18):10145–62. doi: 10.1002/ece3.5501 (PMC6787824; doi:10.1002/ece3.5501)
Supplement: Supplementary file 1 [file ECE3-9-10145-s001.docx]

**8. Supplementary Appendix**

Table S1. Proportion of variance explained by the different axes of the PCA conducted on culmen length, maximum head length, bill depth at the base and bill depth at nostrils representing an index of bill size.

| **Importance of components:** | |  |  |  |
| --- | --- | --- | --- | --- |
|  | **PC1** | **PC2** | **PC3** | **PC4** |
| Standard deviation | 1.91 | 0.44 | 0.25 | 0.22 |
| Proportion of variance | 0.92 | 0.05 | 0.01 | 0.01 |
| Cumulative proportion | 0.92 | 0.97 | 0.98 | 1.00 |

Table S2. Accepted and mean measured (± SD) values of the standard material used in the stable isotope analysis performed in this study, as well as the mean minimum and maximum values obtain. The "n" refers to the number of samples of standards materials used.

| **Standard material name** | ***δ*^15^N_Air_ (‰)** | | | | ***δ*^13^C_VPDB_ (‰)** | | | |
| --- | --- | --- | --- | --- | --- | --- | --- | --- |
|  | **Accepted value** | **Measured values** | | | **Accepted value** | **Measured values** | | |
|  | **Mean ± SD** | **n** | **Mean ± SD** | **Minimum - maximum** | **Mean ± SD** | **n** | **Mean ± SD** | **Minimum - maximum** |
| **IAEA CH6** |  |  |  |  | –10.4 ± 0.0 | 21 | –10.8 ± 0.3 | –11.2 to -10.3 |
| **IAEA CH7** |  |  |  |  | –32.2 ± 0.1 | 22 | –32.2 ± 0.2 | –32.5 to –31.7 |
| **IAEA 600** | +1.0 ± 0.2 | 12 | +0.8 ± 0.1 | 0.6 to 1.0 | –27.8 ± 0.0 | 12 | –27.8 ± 0.4 | –28.2 to –27.2 |
| **USGS 40** | –4.5 ± 0.1 | 24 | –4.6 ± 0.1 | –4.9 to –4.4 | –26.2 ± 0.1 | 24 | –26.5 ± 0.3 | –27.1 to –26.0 |
| **IAEA N1** | +0.4 ± 0.1 | 19 | +0.3 ± 0.3 | 0 to 0.8 |  |  |  |  |
| **IAEA N2** | +20.4 ± 0.1 | 20 | +19.7 ± 0.3 | 19.3 to 20.2 |  |  |  |  |
| **IAEA NO3** | +4.7 ± 0.1 | 9 | +4.4 ± 0.3 | 3.7 to 4.8 |  |  |  |  |
| **USGS 34** | –1.8 ± 0.2 | 9 | –1.9 ± 0.1 | –2.0 to –1.8 |  |  |  |  |

Table S3. Mean values (± SD) of five biometric measurements of three *Calonectris* species: tarsus length, culmen length, maximum head length, bill depth at base and bill depth at nostrils and PC1 scores (as a proxy of bill size). (n) Number of measured Scopoli’s, Cory’s and Cape Verde shearwater individuals.

| **Species** | **n** | | **Tarsus (mm)** | | **Maximum head length (mm)** | | **Culmen length (mm)** | | **Bill depth at base (mm)** | | **Bill depth at nostrils (mm)** | | **PC1 scores** | |
| --- | --- | --- | --- | --- | --- | --- | --- | --- | --- | --- | --- | --- | --- | --- |
|  | **Males** | **Females** | **Males** | **Females** | **Males** | **Females** | **Males** | **Females** | **Males** | **Females** | **Males** | **Females** | **Males** | **Females** |
| Scopoli’s shearwater | 21 | 22 | 55.4 ± 1.8 | 53.1 ± 1.8 | 109.3 ± 3.9 | 102.8 ± 3.9 | 52.2 ± 2.2 | 49.0 ± 2.2 | 19.8 ± 1.5 | 17.3 ± 1.5 | 14.5 ± 1.0 | 12.7 ± 1.0 | -0.6 ± 1.3 | 1.2 ± 1.3 |
| Cory’s shearwater | 44 | 40 | 58.0 ± 2.0 | 56.0 ± 2.1 | 114.1 ± 3.9 | 109.8 ± 3.9 | 55.3 ± 2.3 | 52.5 ± 2.1 | 21.6 ± 1.3 | 19.6 ± 1.4 | 15.7 ± 1.0 | 14.4 ± 1.0 | -2.0 ± 1.3 | -0.6 ± 1.2 |
| Cape Verde shearwater | 7 | 10 | 49.4 ± 1.8 | 46.5 ± 1.8 | 95.0 ± 2.5 | 91.3 ± 3.0 | 45.1 ± 1.8 | 42.7 ± 2.1 | 15.7 ± 1.0 | 14.6 ± 1.0 | 11.9 ± 1.7 | 10.6 ± 1.6 | 2.7 ± 0.8 | 3.8 ± 0.9 |

Table S4. Sexual size dimorphism index (SSI; Storer’s index, see Materials and Methods) in biometric measurements and multivariate (PC1 scores) bill measurements of three *Calonectris* shearwaters.

| **Species** | **n** | **Tarsus** | **Maximum head length** | **Culmen length** | **Bill depth** | **Bill depth at nostril** | **PC1 score** |
| --- | --- | --- | --- | --- | --- | --- | --- |
| Scopoli’s shearwater | 43 | 4.2 | 6.2 | 6.4 | 13.4 | 13.3 | 0.3 |
| Cory’s shearwater | 84 | 3.4 | 3.9 | 5.2 | 9.8 | 8.4 | -1.4 |
| Cape Verde shearwater | 17 | 6.0 | 4.0 | 5.3 | 7.8 | 11.8 | 3.4 |

Table S5. Linear mixed models (LMMs) testing for sex and species effects on seven migratory parameters in the three *Calonectris* species. (a) Structure of the candidate models evaluated to explain our data and values of Akaike’s Information Criterion adjusted for small sample sizes (AICc). The most parsimonious model, and the models with ∆AICc < 2, are shown in bold. (b) Results of the mean estimates (and 95% confidence intervals in parenthesis) obtained from the best models and performing model averaging between the best-supported models when ∆AICc < 2. (c) Relative variance importance of the fixed effects obtained from model averaging. (d) Estimated variance (± SD) of random effects of models in which we did not perform model averaging. All evaluated models included bird identity, year and nonbreeding area as random effects.

|  | **Departure from the breeding colony (Julian date)** | **Days in transit to the nonbreeding areas** | **Total duration of the nonbreeding period (in days)** | **Days in nonbreeding areas** | **Onset of the pre-breeding migration (Julian date)** | **Days in transit to the breeding colony** | **Arrival at the breeding colony (Julian date)** |
| --- | --- | --- | --- | --- | --- | --- | --- |
| **(a) Fixed factors structure (AICc)** | |  |  |  |  |  |  |
| Sex + Species + Sex:Species | 2354.7 | 1868.3 | 2463.4 | 2396.3 | 2152.9 | 1954.3 | 2181 |
| Sex + Species | **2351.1** | 1864.1 | **2459.3** | **2393.0** | **2150.1** | **1952.3** | 2179.4 |
| Sex | 2378.1 | 1878.8 | 2472.3 | 2425.8 | 2158.0 | 1972.3 | **2175.1** |
| Species | **2352.2** | **1862.0** | **2457.5** | **2391.8** | **2148.2** | 1954.6 | 2179.4 |
| Constant | 2378.5 | 1876.7 | 2470.6 | 2425.0 | 2156.0 | 1973.4 | **2175.2** |
| **(b) Fixed effects (Estimates)** | |  |  |  |  |  |  |
| Males | 316.4 (312.5, 320.5) |  | 113.7 (107.6, 119.5) | 61.3 (52.5, 70.2) | 44.6 (41.2, 47.9) | 20.8 (19.0, 22.6) | 65.7 (59.4, 71.8) |
| Females | 321.1 (317.0, 325.2) |  | 111.9 (106.0, 118.1) | 58.9 (50.1, 67.6) | 45.4 (42.1, 48.9) | 23.1 (21.3, 25.0) | 68.3 (62.2, 74.4) |
| Cory’s shearwater | 318.7 (314.8, 322.6) | 14.8 (12.0, 17.5) | 112.8 (107.9, 117.8) | 60.1 (51.4, 68.8) | 45.0 (42.0, 48.0) | 22.0 (20.4, 24.0) |  |
| Scopoli’s shearwater | 298.7 (295.0, 302.4) | 8.8 (7.1, 9.7) | 129.1 (122.9, 135.2) | 79.3 (66.8, 91.8) | 49.2 (46.3, 52.1) | 14.0 (12.0, 16.0) |  |
| Cape Verde shearwater | 320.3 (317.5, 323.9) | 10.9 (9.1, 11.2) | 114.9 (109.5, 118.3) | 80.5 (73.8, 87.2) | 55.0 (51.5, 58.6) | 13.9 (11.7, 16.5) |  |
| **(c) Relative variance importance (%)** | |  |  |  |  |  |  |
| Sex | 0.6 |  | 0.3 | 0.4 | 0.3 |  | 0.5 |
| Species | 1.0 |  | 1.0 | 1.0 | 1.0 |  |  |
| Sex:Species |  |  |  |  |  |  |  |
| **(d) Random effects (variance ± SD)** | |  |  |  |  |  |  |
| Individual |  | 0.0 ± 0.0 |  |  |  | 8.9 ± 3.0 |  |
| Year |  | 4.2 ± 2.1 |  |  |  | 0.7 ± 0.8 |  |
| Nonbreeding area |  | 4.4 ± 2.1 |  |  |  | 0.0 ± 0.0 |  |

Table S6. Linear mixed models (LMMs) testing for sex and species effects on the maximum distance travelled from the colony to the centroids of the nonbreeding areas in the three *Calonectris* species. (a) Structure of the candidate models evaluated to explain our data and values of Akaike’s Information Criterion adjusted for small sample sizes (AICc). The most parsimonious model, and the models with ∆AICc < 2, are shown in bold. (b) Results of the mean estimates (and 95% confidence intervals in parenthesis) obtained from the best models and performing model averaging between the best-supported models when ∆AICc < 2. (c) Relative variance importance of the fixed effects obtained from model averaging. (d)

Estimated variance (± SD) of random effects of models in which we did not perform model averaging. All evaluated models included bird identity, year and nonbreeding area as random effects.

| **Maximum distance travelled from the colony to the centroids of the nonbreeding areas** | | | |
| --- | --- | --- | --- |
| **(a) Fixed factors structure** | **AICc** | **∆AICc** | **AICc_weight_** |
| **Nonbreeding area** | **712.2** | **0.0** | **0.457** |
| **Species + Nonbreeding area** | **713.2** | **1.0** | **0.275** |
| Sex + Nonbreeding area | 714.3 | 2.1 | 0.161 |
| Sex + Species + Nonbreeding area | 715.4 | 3.2 | 0.094 |
| Sex + Species + Nonbreeding area + Sex:Species | 719.6 | 7.3 | 0.012 |
| Sex + Species | 1239.2 | 526.9 | 0.000 |
| Species | 1239.3 | 527.0 | 0.000 |
| Sex + Species + Sex:Species | 1241.9 | 529.7 | 0.000 |
| Constant | 1247.1 | 534.8 | 0.000 |
| Sex | 1247.1 | 534.9 | 0.000 |
| **(b) Fixed effects** | **Estimates** | | |
| Agulhas Current:Cory’s shearwater | 8.1 (7.5, 8.8) | | |
| Benguela Current | -0.7 (-1.0, -0.3) | | |
| Brazil Current | -1.0 (-1.5, -0.4) | | |
| Canary Current | -6.1 (-6.6, -5.7) | | |
| Guinea Current | -3.8 (-4.3, -3.2) | | |
| North Atlantic | -7.3 (-8.1, -6.5) | | |
| South Atlantic | -0.1 (-0.7, 0.5) | | |
| Scopoli’s shearwater | -0.1 (-1.1, 1.0) | | |
| Cape Verde shearwater | -1.2 (-2.3, -0.1) | | |
| **c) Relative variance importance (%)** |  |  |  |
| Nonbreeding area | 1.0 |  |  |
| Species | 0.4 |  |  |

Table S7. Nonbreeding destinations of males and females of Cory’s shearwater separated by breeding colony.

| **Breeding colony** | **Nonbreeding area** | **n** | |
| --- | --- | --- | --- |
|  |  | **Males** | **Females** |
| Vila islet (Azores) | Agulhas Current | 0 | 5 |
|  | Benguela Current | 7 | 0 |
|  | Brazil Current | 1 | 2 |
|  | North Atlantic Ocean | 7 | 0 |
|  | South Atlantic Ocean | 1 | 2 |
| Montaña Clara (Canary Islands) | Agulhas Current | 0 | 2 |
|  | Benguela Current | 7 | 9 |
|  | Brazil Current | 0 | 5 |
|  | Canary Current | 1 | 0 |
|  | South Atlantic Ocean | 2 | 0 |
| Veneguera (Canary Islands) | Agulhas Current | 9 | 7 |
|  | Benguela Current | 64 | 50 |
|  | Brazil Current | 1 | 5 |
|  | Canary Current | 13 | 10 |
|  | South Atlantic Ocean | 3 | 2 |

Table S8. Sizes of wintering core areas (km^2^) for males and females of Scopoli’s, Cory’s and Cape Verde shearwaters grouped by nonbreeding area and year (minimum number of birds = 4). Core area sizes were calculated based on the 50% UD kernel contour for each sex, nonbreeding area and year.

| **Species** | **Nonbreeding area** | **Year** | **Core area (km^2^)** | | | |
| --- | --- | --- | --- | --- | --- | --- |
|  |  |  | n Males | **Males** | n Females | **Females** |
| Scopoli’s shearwater | Canary Current | 2011 | 6 | 569,564 | 7 | 701,080 |
|  |  | 2012 | 9 | 421,943 | 4 | 488,317 |
|  | Guinea Current | 2012 | 6 | 1,308,328 | 5 | 1,465,092 |
|  | Benguela Current | 2010 | 10 | 711,411 | 5 | 953,732 |
|  | Mean |  |  | **752,812** |  | **902,055** |
| Cory’s shearwater | Canary Current | 2012 | 5 | 467,507 | 4 | 704,844 |
|  | Benguela Current | 2008 | 9 | 1,141,296 | 8 | 1,279,964 |
|  |  | 2009 | 11 | 562,722 | 6 | 628,410 |
|  |  | 2010 | 8 | 769,852 | 10 | 759,279 |
|  |  | 2011 | 18 | 178,743 | 5 | 466,224 |
|  |  | 2012 | 22 | 994,756 | 21 | 1,027,324 |
|  |  | 2013 | 9 | 651,885 | 6 | 647,138 |
|  | Mean |  |  | **680,966** |  | **787,598** |
| Cape Verde shearwater | Brazil Current | 2010 | 4 | 632,719 | 5 | 613,933 |
|  |  | 2011 | 4 | 526,576 | 4 | 438,259 |
|  | Mean |  |  | **579,648** |  | **526,096** |

Table S9. Linear mixed models (LMMs) testing for potential effects of sex and species on the mean size of wintering core areas (calculated based on the 50% UD kernel contour for each sex, nonbreeding area and year) of the three *Calonectris* species during the nonbreeding period. (a) Structure of the candidate models evaluated to explain our data and their associated measures of information (AICc: corrected Akaike’s Information Criterion; ΔAICc: AICc increments of each model in comparison with the best model; AICc_weight_: AICc weights of each model in relation to the set of candidate models). The most parsimonious model is shown in bold. (b) Mean estimates (and 95% confidence intervals in parenthesis) of the fixed effects. (c) Variance (± SD) and random variance explained (calculated as the percentage of the variance of each random effect divided by the total variance explained by all random effects) by the random effects. All evaluated models included year and nonbreeding area as random effects.

| **Mean size of wintering core areas** | | | | |
| --- | --- | --- | --- | --- |
| **(a) Fixed factors structure** | **AICc** | **∆AICc** | **AICc_weight_** | |
| Sex | **887.8** | **0.0** | **0.592** | |
| Constant | 890.1 | 2.3 | 0.192 | |
| Sex + Species | 890.6 | 2.7 | 0.150 | |
| Species | 892.8 | 4.9 | 0.050 | |
| Sex + Species + Sex:Species | 895.1 | 7.3 | 0.015 | |
| **(b) Fixed effects** | **Estimates** | | | |
| Males | 806.5 (553.6, 1059.5) | | | |
| Females | 618.6 (365.7, 871.6) | | | |
| **(c) Random Effects** | **Variance ± SD** | | | **Random variance explained (%)** |
| Nonbreeding area | 70478 ± 265.5 | | | 38.6 |
| Year | 1627 ± 40.3 | | | 0.9 |
| Residuals | 110596 ± 332.6 | | | 60.5 |

Table S10. Spatial overlap of the general use and wintering core areas of males and females of the three *Calonectris* species. Sizes of the general use and wintering core areas were calculated based on the 95% and 50% KUD contour, respectively, for each sex, nonbreeding area and year (minimum number of birds = 4).

| **Species** | **Nonbreeding area** | **Year** | **Overlap** | |
| --- | --- | --- | --- | --- |
|  |  |  | **95% KUD** | **50% KUD** |
| Scopoli’s shearwater | Canary Current | 2011 | 0.776 | 0.860 |
|  |  | 2012 | 0.734 | 0.908 |
|  | Guinea Current | 2012 | 0.460 | 0.235 |
|  | Benguela Current | 2010 | 0.750 | 0.953 |
| Cory’s shearwater | Canary Current | 2012 | 0.987 | 0.963 |
|  | Benguela Current | 2008 | 0.902 | 0.718 |
|  |  | 2009 | 0.452 | 0.281 |
|  |  | 2010 | 0.811 | 0.581 |
|  |  | 2011 | 0.475 | 0.763 |
|  |  | 2012 | 0.644 | 0.931 |
|  |  | 2013 | 0.894 | 0.786 |
| Cape Verde shearwater | Brazil Current | 2010 | 0.845 | 0.965 |
|  |  | 2011 | 0.831 | 0.733 |

Table S11. Linear mixed models (LMMs) testing for potential effects of sex and species on the night flight index (NFI) of the three *Calonectris* species during the nonbreeding period. (a) Structure of the candidate models evaluated to explain our data and their associated measures of information (AICc: corrected Akaike’s Information Criterion; ΔAICc: AICc increments of each model in comparison with the best model; AICc_weight_: AICc weights of each model in relation to the set of candidate models). The most parsimonious model, and the models with ∆AICc < 2, are shown in bold. (b) Results of the mean estimates (and 95% confidence intervals in parenthesis) obtained when performing model averaging between the best-supported models with ∆AICc < 2. (c) Relative variance importance of the fixed effects obtained from model averaging. All evaluated models included bird identity and year as random effects.

| **NFI** | | | |
| --- | --- | --- | --- |
| **(a) Fixed factors structure** | **AICc** | **∆AICc** | **AICc_weight_** |
| **Sex + Species + Sex:Species** | **-103.8** | **0.0** | **0.501** |
| **Sex + Species** | **-102.9** | **0.9** | **0.317** |
| Species | -101.8 | 2.0 | 0.181 |
| Constant | -73.4 | 30.4 | 0.000 |
| Sex | -73.3 | 30.6 | 0.000 |
| **(b) Fixed effects** | **Estimates** | | |
| Males:Cory’s shearwater (Intercept) | -0.3 (-0.4, -0.2) | | |
| Females:Cory’s shearwater | -0.4 (-0.5, -0.3) | | |
| Scopoli’s shearwater | -0.3 (-0.4, -0.2) | | |
| Cape Verde shearwater | -0.2 (-0.4, -0.1) | | |
| Females:Scopoli’s shearwater | 0.1 (0.0, 0.3) | | |
| Females:Cape Verde shearwater | 0.0 (-0.2, 0.2) | | |
| **(c) Relative variance importance (%)** |  | | |
| Sex | 1.0 | | |
| Species | 1.0 | | |
| Sex:Species | 0.6 | | |

Table S12. Post-hoc pairwise comparisons based on the difference between the least square means (and 95% confidence intervals in parenthesis) of the parameters of the most parsimonious models testing the effects of sex, species and bill size on δ^13^C (A) and δ^15^N (B) values of the S13 remige. Statistically significant results marked in bold are in relation to the significance level calculated using the Bonferroni correction (*P* < 0.002). All the models included bird identity, year and nonbreeding area as random effects.

| **A) δ^13^C** | | |
| --- | --- | --- |
| **(a) Pairwise comparison** | **Estimates** | ***P*** |
| Males – Females | 0.6 (0.3, 0.9) | **<0.001** |
| Cory’s – Scopoli’s shearwater | -0.2 (-0.7, 0.3) | 0.369 |
| Cory’s – Cape Verde shearwater | -0.4 (-1.4, 0.5) | 0.359 |
| Scopoli’s – Cape Verde shearwater | -0.2 (-1.0, 0.6) | 0.580 |
| Males Cory’s – Females Cory’s | 0.2 (-0.2, 0.6) | 0.272 |
| Males Cory’s – Males Scopoli’s | -0.7 (-1.2, -0.2) | 0.006 |
| Males Cory’s – Females Scopoli’s | 0.4 (-0.3, 1.2) | 0.223 |
| Males Cory’s – Males Cape Verde | -0.6 (-1.6, 0.5) | 0.298 |
| Males Cory’s – Females Cape Verde | -0.1 (-1.3, 1.0) | 0.811 |
| Females Cory’s – Males Scopoli’s | -0.9 (-1.3, -0.4) | **<0.001** |
| Females Cory’s – Females Scopoli’s | 0.2 (-0.3, 0.8) | 0.401 |
| Females Cory’s – Males Cape Verde | -0.8 (-1.6, 0.1) | 0.082 |
| Females Cory’s – Females Cape Verde | -0.3 (-1.3, 0.6) | 0.480 |
| Males Scopoli’s – Females Scopoli’s | 1.1 (0.7, 1.5) | **<0.001** |
| Males Scopoli’s – Males Cape Verde | 0.1 (-0.8, 1.0) | 0.788 |
| Males Scopoli’s – Females Cape Verde | 0.5 (-0.5, 1.5) | 0.293 |
| Females Scopoli’s – Males Cape Verde | -1.0 (-1.8, -0.2) | 0.011 |
| Females Scopoli’s – Females Cape Verde | -0.6 (-1.4, 0.2) | 0.164 |
| Males Cape Verde – Females Cape Verde | 0.4 (-0.1, 0.9) | 0.112 |

| **B) δ^15^N** | | |
| --- | --- | --- |
| **(a) Pairwise comparison** | **Estimates** | ***P*** |
| Males – Females | 0.9 (0.5, 1.4) | **<0.001** |
| Cory’s – Scopoli’s shearwater | 0.6 (-0.2, 1.3) | 0.122 |
| Cory’s – Cape Verde shearwater | -4.3 (-5.8, -2.9) | **<0.001** |
| Scopoli’s – Cape Verde shearwater | -4.9 (-6.2, -3.7) | **<0.001** |
| Males Cory’s – Females Cory’s | 0.4 (-0.1, 1.0) | 0.106 |
| Males Cory’s – Males Scopoli’s | 0.2 (-0.5, 1.0) | 0.510 |
| Males Cory’s – Females Scopoli’s | 1.4 (0.3, 2.4) | 0.013 |
| Males Cory’s – Males Cape Verde | -4.7 (-6.3, -3.1) | **<0.001** |
| Males Cory’s – Females Cape Verde | -3.5 (-5.3, -1.8) | **<0.001** |
| Females Cory’s – Males Scopoli’s | -0.2 (-0.9, 0.5) | 0.568 |
| Females Cory’s – Females Scopoli’s | 0.9 (0.1, 1.8) | 0.036 |
| Females Cory’s – Males Cape Verde | -5.1 (-6.4, -3.9) | **<0.001** |
| Females Cory’s – Females Cape Verde | -4.0 (-5.4, -2.5) | **<0.001** |
| Males Scopoli’s – Females Scopoli’s | 1.1 (0.5, 1.7) | **<0.001** |
| Males Scopoli’s – Males Cape Verde | -5.0 (-6.3, -3.6) | **<0.001** |
| Males Scopoli’s – Females Cape Verde | -3.8 (-5.3, -2.2) | **<0.001** |
| Females Scopoli’s – Males Cape Verde | -6.1 (-7.2, -4.9) | **<0.001** |
| Females Scopoli’s – Females Cape Verde | -4.9 (-6.1, -3.6) | **<0.001** |
| Males Cape Verde – Females Cape Verde | 1.2 (0.4, 1.9) | **0.002** |

Table S13. Bayesian estimates of the standard ellipse areas (SEAb, ‰^2^) ± SD (in parenthesis) for the values of δ^13^C and δ^15^N of the S13 remige of males and females of the three *Calonectris* species for each nonbreeding area and year (we only considered nonbreeding areas containing a minimum of 4 birds per sex and year).

| **Species** | **Nonbreeding area** | **Year** | **SEAb** | | | |
| --- | --- | --- | --- | --- | --- | --- |
|  |  |  | **Males (‰^2^)** | n | **Females (‰^2^)** | n |
| Scopoli’s shearwater | Guinea Current | 2011 | 1.6 (0.7, 4.2) | 6 | 0.5 (0.2, 1.2) | 7 |
|  | Canary Current | 2011 | 0.8 (0.4, 1.7) | 9 | 1.1 (0.4, 3.8) | 4 |
|  |  | 2012 | 0.1 (0.0, 0.2) | 6 | 0.5 (0.2, 1.6) | 5 |
|  | Benguela Current | 2010 | 0.8 (0.4, 1.5) | 10 | 1.2 (0.4, 3.4) | 5 |
| Cory’s shearwater | Canary Current | 2012 | 0.4 (0.2, 1.4) | 5 | 0.1 (0.0, 0.3) | 4 |
|  | Benguela Current | 2008 | 0.2 (0.1, 0.5) | 9 | 0.7 (0.3, 1.7) | 8 |
|  |  | 2009 | 0.7 (0.4, 1.3) | 11 | 0.4 (0.2, 1.1) | 1.1 |
|  |  | 2010 | 0.7 (0.3, 1.5) | 8 | 1.1 (0.6, 2.1) | 10 |
|  |  | 2011 | 1.7 (1.1, 2.8) | 18 | 0.7 (0.3, 2.0) | 5 |
|  |  | 2012 | 1.5 (1.0, 2.4) | 22 | 1.6 (1.1, 2.6) | 21 |
|  |  | 2013 | 0.4 (0.2, 0.9) | 9 | 1.3 (0.6, 3.5) | 6 |
| Cape Verde shearwater | Brazilian Current | 2010 | 0.5 (0.2, 1.6) | 4 | 0.2 (0.1, 0.6) | 5 |
|  |  | 2011 | 0.4 (0.2, 1.7) | 4 | 0.6 (0.2, 2.0) | 4 |

Table S14. Linear mixed models (LMMs) testing for sex, species and the size of the core area of wintering distribution effects on the Bayesian estimate of the standard ellipse area (SEAb) used as an approach to characterize the isotope niche widths (INW) of the three *Calonectris* species. (a) Structure of the candidate models evaluated to explain our data and their associated measures of information (AICc: corrected Akaike’s Information Criterion; ΔAICc: AICc increments of each model in comparison with the best model; AICc_weight_: AICc weights of each model in relation to the set of candidate models). The most parsimonious model, and the models with ∆AICc < 2, are shown in bold. (b) Results of the mean estimates (and 95% confidence intervals in parenthesis) with adjusted SE obtained after performing model averaging between the best-supported models with ∆AICc < 2. (c) Relative variance importance of the fixed effects obtained from model averaging. All evaluated models included nonbreeding area and year as random effects.

| **INW** |  |  |  |
| --- | --- | --- | --- |
| **(a) Fixed factors structure** | **AICc** | **∆AICc** | **AICc_weight_** |
| **Constant** | **91.7** | **0.0** | **0.405** |
| **Species** | **92.9** | **1.2** | **0.227** |
| Core area | 94.0 | 2.3 | 0.130 |
| Sex | 94.4 | 2.7 | 0.106 |
| Core area + Species | 96.0 | 4.3 | 0.047 |
| Sex + Species | 96.1 | 4.3 | 0.047 |
| Core area + Sex | 97.0 | 5.2 | 0.030 |
| Core area + Sex + Species | 99.6 | 7.8 | 0.008 |
| Sex + Species + Sex:Species | 103.7 | 11.9 | 0.001 |
| Core area + Sex + Species + Sex:Species | 108.0 | 16.3 | 0.000 |
| **(b) Fixed effects** | **Estimates** | | |
| Core area | 1.0 (0.7, 1.4) | | |
| Cory’s shearwater | 0.8 (0.1, 1.4) | | |
| Scopoli’s shearwater | 1.5 (0.9, 2.0) | | |
| Cape Verde shearwater | 0.4 (-0.6, 1.4) | | |
| **c) Relative variance importance (%)** |  | | |
| Sex | 0.1 |  |  |
| Species | 0.4 |  |  |
| Core area | 0.2 |  |  |
